# Low maternal vitamin A intake increases the incidence of teratogen induced congenital diaphragmatic hernia in mice

**DOI:** 10.1038/s41390-021-01409-6

**Published:** 2021-03-02

**Authors:** Ayanna W. Rocke, Tianna G. Clarke, Timothy R. A. Dalmer, Sydney A. McCluskey, Juan F. Garcia Rivas, Robin D. Clugston

**Affiliations:** 1 Department of Physiology, Faculty of Medicine and Dentistry, University of Alberta, Edmonton, AB Canada; 2 Women and Children’s Health Research Institute, University of Alberta, Edmonton, AB Canada

## Abstract

**Background:**

Congenital diaphragmatic hernia (CDH) is a severe birth defect associated with high perinatal mortality and long-term morbidity. The etiology of CDH is poorly understood although abnormal retinoid signaling has been proposed to contribute to abnormal diaphragm development. Existing epidemiological data suggest that inadequate dietary vitamin A intake is a risk factor for developing CDH.

**Methods:**

Using a mouse model of teratogen-induced CDH, the objective of this study was to test the hypothesis that low maternal vitamin A intake contributes to abnormal diaphragm development. To test this hypothesis, we optimized a model of altered maternal dietary vitamin A intake and a teratogenic model of CDH in mice that recapitulates the hallmark features of posterolateral diaphragmatic hernia in humans.

**Results:**

Our data uniquely show that low maternal dietary vitamin A intake and marginal vitamin A status increases the incidence of teratogen-induced CDH in mice.

**Conclusion:**

Low dietary vitamin A intake and marginal vitamin A status lead to an increased incidence of teratogen-induced CDH in mice, highlighting the importance of adequate dietary vitamin A intake and CDH risk.

**Impact:**

- This study describes and validates a mouse model of altered maternal and fetal vitamin A status.
- This study links existing epidemiological data with a mouse model of teratogen-induced congenital diaphragmatic hernia, highlighting the importance of low maternal vitamin A intake as a risk factor for the development of congenital diaphragmatic hernia.
- This study supports the *Retinoid Hypothesis*, which posits that the etiology of congenital diaphragmatic hernia is linked to abnormal retinoid signaling in the developing diaphragm.

## Introduction

Congenital diaphragmatic hernia (CDH) is a frequently occurring, life-threatening birth defect. It is characterized by incomplete formation of the diaphragm and severe pulmonary hypoplasia secondary to herniation of the abdominal contents into the thorax.^1^ The etiology of CDH is poorly understood and is associated with a large number of genetic defects, but no clear etiologic driver.^1,2^ One of the leading hypotheses explaining the development of CDH is the *retinoid hypothesis*. As originally proposed by Greer et al.,^3^ this hypothesis states that abnormal retinoid signaling early in diaphragm development contributes to the etiology of CDH. In this context, the term retinoid refers to vitamin A and its metabolites, including the potent signaling molecule retinoic acid.^4^ While the retinoid hypothesis was posited to explain the origins of abnormal diaphragm development in CDH, it is also consistent with the *dual-hit hypothesis* of pulmonary hypoplasia in CDH. This hypothesis posits that pulmonary hypoplasia in CDH arises from two insults, one directly affecting the lungs early in development and one secondary to abnormal diaphragm development and abdominal organ herniation.^5^ With a well-described role in lung development,^6,7^ abnormal retinoic acid signaling may contribute to both abnormal lung and diaphragm development in CDH.

The retinoid hypothesis was primarily based on experimental evidence from rodent studies, as well as some insightful human data.^3^ Data from rodent studies included the occurrence of CDH in the offspring of vitamin A-deficient rats,^8–10^ diaphragm defects in retinoic acid receptor knock-out mice,^11^ the fact that the CDH-inducing teratogen nitrofen inhibits retinoic acid synthesis,^12^ and that nitrofen-induced CDH could be rescued by co-administration of large doses of vitamin A.^13,14^ In terms of human CDH, it was reported that newborns with CDH had decreased circulating markers of vitamin A status.^15^ Since being proposed in 2003, several studies have supported the retinoid hypothesis, including contributions from our group^16–20^ and others.^21–25^ Most recently, Michikawa et al.^26^ reported that maternal dietary vitamin A intake in the first trimester was inversely associated with CDH. This finding was consistent with a previous human study concluding that low maternal vitamin A intake during pregnancy increased the risk of CDH.^27^ While both of these studies were limited by the small number of CDH cases included, they are supportive of the retinoid hypothesis.

In this study we aimed to explore the link between maternal vitamin A intake and CDH risk using a mouse model of teratogen-induced CDH. The rationale for this study was based on the epidemiological link between low maternal vitamin A intake and increased CDH risk,^26,27^ leading us to hypothesize that low maternal vitamin A intake increases the risk of teratogen-induced CDH in mice. We aimed to test this hypothesis in a mouse model of teratogen-induced CDH, using altered dietary vitamin A intake to manipulate maternal vitamin A status and susceptibility to CDH. We expected that low maternal vitamin A intake would lead to an increase in the incidence of teratogen-induced CDH, and that excess maternal vitamin A intake would lead to a reduced incidence of CDH. While our primary outcome measure was the incidence of CDH, we also assessed the severity of CDH and the co-occurrence of other gross abnormalities. Here, we describe a mouse model of teratogen-induced CDH with an alteration in maternal vitamin A status, and report that marginal maternal vitamin A status increases the incidence of teratogen-induced CDH in mice.

## Materials and methods

### Animals and breeding

All mouse studies were approved by the University of Alberta Animal Research Ethics Committee and performed in accordance with the guidelines established by The Canadian Council on Animal Care. All experiments were conducted using BALB/c mice (The Jackson Laboratory, Bar Harbor, ME, USA), housed in the University of Alberta’s conventional animal facility. Animals were housed in individually ventilated cages with a 12-h light–dark cycle and a temperature-controlled environment (21 ± 2 °C). To obtain timed-pregnant mice, female mice were transferred to male cages between 1500 and 1800 and left overnight. The following morning between 0800 and 0900 the female genital region was examined for the presence of a vaginal sperm plug as a positive sign of copulation. If a vaginal sperm plug was present, midday of that day was considered gestational day 0.5.

### Dietary vitamin A manipulation

Purified rodent diets were purchased from Bio-Serv (Flemington, NJ, USA). Diets were custom made with three differing vitamin A levels: 0 IU/g vitamin A, 4 IU/g vitamin A, and 25 IU/g vitamin A. Other than the differing vitamin A content, the purified diets were identical and used a standard macronutrient composition with an AIN-93G vitamin and mineral mix. At the time of weaning, female mice were randomly assigned to an experimental diet containing 0, 4, or 25 IU/g vitamin A for 3 months. At the end of this period, mice were maintained on their respective purified diet and used for timed-pregnant mating as described above. Throughout this experimental time course, mice were weighed on a weekly basis.

### Teratogen administration

To induce CDH, timed-pregnant mice were treated with a teratogenic combination of nitrofen (2,4‐dichlorophenyl 4‐nitrophenyl ether; China National Chemical Construction Jiangsu Company, Nanjing, China), and bisdiamine ([dichloroacetyl]-1,8-octamethylenediamine; MP Biomedicals, Solon, OH, USA). Note, while administration of nitrofen is a well-established model of CDH in rats, it is relatively ineffective in mice.^28,29^ Here we use a teratogenic combination of nitrofen and bisdiamine in mice based on previous descriptions,^17,30^ and emphasize that both compounds have previously been shown to inhibit retinoic acid synthesis in vitro and block retinoic acid signaling in vivo.^12,17^ This teratogenic combination was prepared immediately before administration by dissolving the nitrofen and bisdiamine in olive oil using a combination of vortex mixing and sonication for ~10 min. Prior to administration, timed-pregnant mice were weighed, and the teratogenic mixture was administered by oral gavage at a concentration of 0.5 g/kg nitrofen and 0.125 g/kg bisdiamine. All pregnant mice were given this teratogenic combination between 1200 and 1300 on gestational day 8.

### Tissue collection

At gestational day 18.5 mice were euthanized by isoflurane inhalation followed by cervical dislocation. An abdominal incision was then made, and the entire gravid uterus was removed and placed in ice-cold phosphate-buffered saline (PBS), prior to further dissection. At this time maternal liver samples were collected and immediately snap frozen in liquid nitrogen. Maternal blood was also collected and subsequently centrifuged for 10 min to separate out the plasma, which was transferred to a clean tube and snap frozen in liquid nitrogen. Liver and plasma samples were then transferred to storage at −80 °C prior to further analysis. The isolated uterus was dissected using a stereo microscope (Stemi 508; Zeiss, Oberkochen, Germany), and the number of fetuses and visible resorptions were recorded. Individual fetuses were then dissected from the uterus, fetal crown–rump length (CRL) was measured, and gross abnormalities were recorded if present. For the purposes of retinoid quantification, fetal liver was removed, snap frozen using liquid nitrogen, and held at −80 °C until high-performance liquid chromatography (HPLC) analysis. For the purposes of determining CDH incidence and severity, fetuses were cut down and their trunk was fixed overnight in 10% buffered formalin at 4 °C. The following day, fetuses were dissected using a stereo microscope and the presence, location, and severity of any diaphragm defects were recorded. All dissected diaphragms were archived in 10% buffered formalin and documented using an Axiocam 105 stereo microscope camera (Zeiss). When collecting samples for vitamin A analysis by HPLC, all tissue collections were carried out in a darkened lab under yellow light, to prevent the degradation of light-sensitive retinoids.

### Diaphragm defect severity

The incidence of CDH was determined by the presence or absence of a hole in the diaphragm. In addition to the occurrence of CDH, we also assessed the size of the diaphragm defect. Indeed, it is well-established in human CDH that the size of the diaphragm defect is an important indicator of clinical outcome.^31^ For human CDH, Tsao and Lally^32^ proposed a universal grading system for diaphragm defect size, including four size classifications. In this system, A grade defects were small and surrounded by muscle, B grade defects were small and involved <50% of the hemidiaphragm, C grade defects were large and included >50% of the hemidiaphragm, and the D grade included absence of the hemidiaphragm.^31,32^ To emulate this grading system, we derived a similar scheme using the same criteria but translated to the mouse diaphragm (Fig. 2g). To visualize the overall occurrence of diaphragm defects heat maps were generated as previously described.^33^ Heat maps were based on the occurrence of different hernia grades and mapped onto a representative diaphragm outline.

### Tissue HPLC analysis

Standard HPLC methods were used to measure tissue retinol and retinyl ester concentrations in maternal plasma and liver, as well as fetal liver samples, as previously described.^34^ In brief, liver samples were homogenized in PBS and then extracted in hexane, whereas plasma samples were directly extracted into hexane. To quantify the concentration of retinol and retinyl ester in our samples, retinyl acetate (MilliporeSigma, St Louis, MO, USA) was used as an internal standard. Following hexane extraction, samples were blown down with nitrogen gas and re-dissolved in mobile phase (70% v/v acetonitrile, 15% v/v methanol, and 15% v/v methylene chloride; MilliporeSigma). Sample analysis was performed using an Agilent 1200 HPLC system (Santa Clara, CA, United States) with a Zorbax Eclipse Plus C18 separating column (4.6 × 250 mm, 5 μm particle size; Agilent). A diode array detector (Agilent) measured peak absorbance at 325 nm and the area under the curve was used to calculate tissue retinol and retinyl ester concentration, factoring in the recovery of the internal standard.

### Statistics

All data were compiled in Excel (Microsoft Corporation, Redmond, WA, USA) and analyzed using Prism 7 (GraphPad, San Diego, CA, USA). Data are presented as the mean ± standard deviation (SD). Statistical analysis depended on the data being analyzed. For our continuous data, when comparing two groups, a Student’s *t*-test was used and when comparing three groups a one-way ANOVA was used with a Tukey’s multiple comparison post-test. For categorical data, contingency tables were constructed (3 × 2) and analyzed using a *Χ*^2^ test for trend, with partitioning into 2 × 2 contingency tables analyzed using Fishers exact test to test for significance between subgroups. In all cases, a *p* value < 0.05 was considered statistically significant.

## Results

### Manipulation of dietary vitamin A intake alters maternal and fetal vitamin A status

**Fig. 1:**
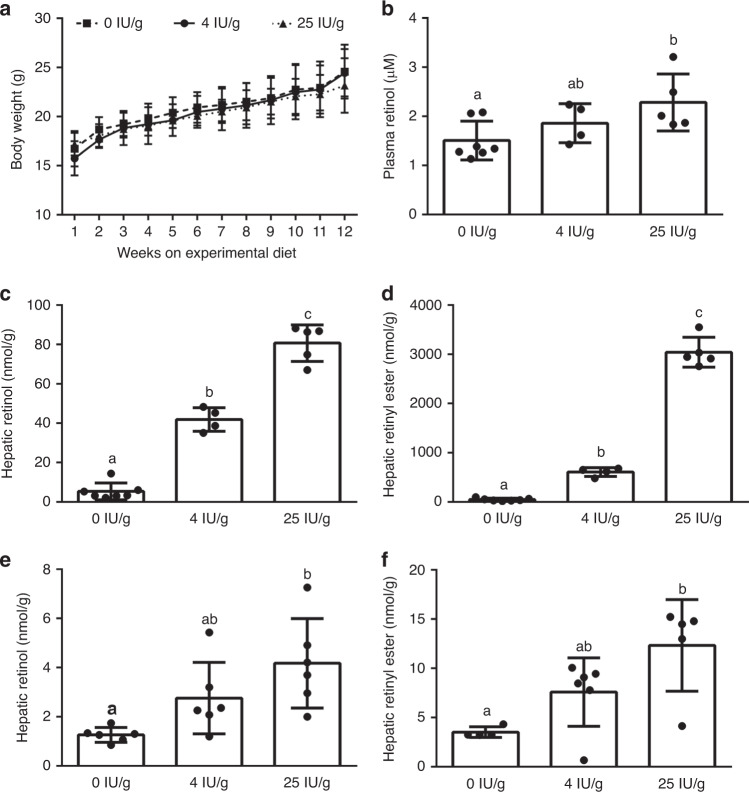
Manipulation of dietary vitamin A intake alters maternal and fetal vitamin A status. The body weight of mice consuming diets with 0, 4, or 25 IU/g vitamin A was not different throughout the 3-month experimental time period (**a**; *n* = 8–18). The plasma retinol (**b**), hepatic retinol (**c**), and hepatic retinyl ester (**d**) concentrations were significantly different in adult female mice, with the lowest levels reported in mice consuming a diet with 0 IU/g vitamin A (*n* = 4–7). In the corresponding fetal mice, hepatic retinol (**e**) and retinyl ester (**f**) concentrations also significantly differed, with the lowest levels recorded in the offspring of mice consuming a diet with 0 IU/g vitamin A (*n* = 4–6). Data plotted as mean ± SD. All data analyzed by one-way ANOVA, with a Tukey’s multiple comparison post-test. Experimental groups that do not share a common letter are significantly different (*p* < 0.05).

To determine the effect of altered maternal vitamin A intake and vitamin A status on the susceptibility to teratogen-induced CDH, we developed a model of altered dietary maternal vitamin A intake and validated its impact on maternal and fetal vitamin A status (Fig. 1). In our validation study, female mice were split into three groups and maintained on experimental diets containing 0, 4, and 25 IU/g vitamin A, respectively. Female mice were placed on experimental diets at the time of weaning for a period of 3 months, they were then bred with male mice and both maternal and fetal tissues were collected at gestational day 18.5. The body weight of experimental mice throughout the 3-month dietary intervention was not different between groups (Fig. 1a). Circulating levels of retinol can be used as a marker of vitamin A status.^35^ Circulating retinol levels were significantly different between experimental groups (Fig. 1b). Plasma retinol levels in mice consuming a diet with 25 IU/g of vitamin A were significantly higher than animals consuming a diet with 0 IU/g vitamin A. There was no significant difference between mice consuming 4 IU/g vitamin A and either group consuming 0 or 25 IU/g vitamin A. As the major storage organ for vitamin A, hepatic vitamin A levels are considered the best marker of whole-body vitamin A status.^35^ Maternal hepatic retinol (Fig. 1c) and retinyl ester (Fig. 1d) levels reflected the vitamin A content of the diet, with significant differences observed between all groups for both parameters. Mice consuming 0 IU/g vitamin A had the lowest level of hepatic retinol, whereas the group consuming 4 IU/g vitamin A had significantly higher retinol levels, and the group consuming 25 IU/g vitamin A had significantly higher levels of retinol than both other groups (Fig. 1c). A similar pattern was observed for hepatic retinyl ester levels, with significantly lower levels of retinyl ester in the level of the 0 IU/g vitamin A group compared to the 4 and 25 IU/g vitamin A group (Fig. 1d). Previous research has indicated that altering dietary vitamin A intake in pregnant animals can also alter fetal tissue vitamin A levels.^36^ In agreement with this, our analysis of hepatic retinoid content in the livers of fetal mice collected at gestational day 18.5 show fetal hepatic retinoid levels tracked maternal dietary vitamin A intake. Specifically, liver retinol (Fig. 1e) and retinyl ester (Fig. 1f) levels were significantly lower in fetuses collected from dams consuming a 0 IU/g vitamin A diet, compared to dams consuming 25 IU/g vitamin A. These data show that consumption of a diet with 0 IU/g vitamin A for 3 months prior to breeding produces a marginal vitamin A status in these animals, as evidenced by low hepatic retinoid stores with maintained circulating retinol levels. Based on these data we used the following designations for our experimental groups: relative to their vitamin A status, the mice consuming the 0 IU/g vitamin A diet were considered to be *marginal*, the mice consuming the 4 IU/g vitamin A diet were considered to be *sufficient*, and the mice consuming the 25 IU/g vitamin A diet were considered to be *excess*.

### Mice with altered vitamin A status do not spontaneously develop CDH

Early studies into the effects of frank vitamin A deficiency in rodents reported multiple congenital anomalies, including the presence of diaphragm defects.^8–10^ Given that our animal model included mice consuming a diet with no vitamin A (0 IU/g vitamin A) to generate dams with a marginal vitamin A status, it was important to establish that the offspring of these animals did not spontaneously develop CDH. As shown in Table 1, we observed no diaphragmatic hernia in the offspring of an extensive series of control mice with marginal, sufficient, and excess vitamin A status. In addition to the absence of diaphragmatic hernia, no other gross abnormalities were observed in any group.

**Table 1:** Mice with altered vitamin A status do not spontaneously develop CDH.

	Maternal vitamin A status		
	Marginal	Sufficient	Excess
Maternal age (days)^a^	131 ± 32	134 ± 29	116 ± 19
Litters examined (*n*)	6	6	7
Litter size (*n*)^a^	5.6 ± 1.6	7.3 ± 1.8	6.8 ± 1.8
Fetuses examined (*n*)	34	44	47
Resorptions (*n*)	0	0	0
Diaphragmatic hernia (*n*)	0	0	0

^a^Data presented as mean ± SD.

### A mouse model of teratogen-induced CDH

**Fig. 2:**
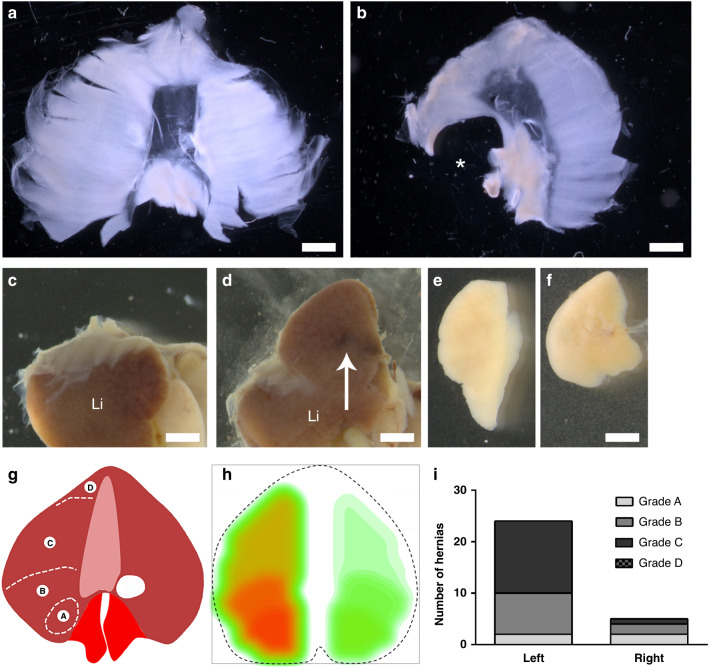
A mouse model of teratogen-induced CDH. Representative images from control and teratogen-treated fetal mice are presented. A normal diaphragm dissected from a control mouse is shown (**a**). Teratogen treatment was associated with the occurrence of diaphragm defects, characterized by a hole in the posterolateral region of the diaphragm (*; **b**). An image of a partially dissected fetus from a control mouse shows the diaphragm sitting atop the liver (Li, **c**). Teratogen treatment was associated with the protrusion of the fetal liver through the hole in the diaphragm (arrow; **d**). The left lung from a control (**e**) and teratogen-treated (**f**) fetus is also shown. Diaphragm defect severity was graded according to a system extrapolated from human CDH (**g**) and was used to generate a heat map of hernia occurrence in teratogen treated mice (**h**). The grade and sidedness of diaphragm defects is also presented (**i**). Scale bars: **a**–**d** = 1 mm, **e**, **f** = 500 µm.

The nitrofen model of teratogen-induced CDH in rats has been one of the mainstays of basic research into the pathogenesis and etiology of CDH since its description over 30 years ago.^29,37,38^ Here we optimize a mouse model of teratogen-induced CDH in animals consuming a regular breeder chow (vitamin A content = 15 IU/g). In these studies, we administered a teratogenic dose of nitrofen and bisdiamine at gestational day 8, modifying previously reported use of this teratogenic combination in mice.^17,30^ Note, administration of olive oil alone (vehicle) did not induce CDH (data not shown). Further, during preliminary studies we found that the dose of bisdiamine had to be carefully titrated; while higher doses produced a higher incidence of CDH, it was also associated with an impractical increased resorption rate (data not shown). The dose used in these studies was chosen to reproducibly generate offspring with CDH, without excess fetal resorption. The ability of this teratogenic combination to induce CDH in the offspring of pregnant mice is described in Table 2. In summary, from a total of 10 litters examined, we observed a total of 26 diaphragmatic hernias, equivalent to an incidence of ~50%. The phenotype of these diaphragm defects was comparable with the nitrofen model in rats and Bochdalek CDH in humans;^18^ Fig. 2). Observed defects were characteristically located in the posterolateral diaphragm (Fig. 2b), associated with herniation of the liver into the thoracic cavity (Fig. 2d), and reduced size of the ipsilateral lung (Fig. 2f). Assessment of defect location and size showed that diaphragm defects were consistently located in the posterolateral region of the left hemidiaphragm (Fig. 2h) and that over 75% of hernias were grade B or C (Fig. 2i). Note, in addition to diaphragm defects, teratogen exposed fetuses also exhibited the presence of facial dysmorpholgies (i.e. median facial clefting) and generalized edema, which is consistent with previously reported effects of nitrofen and bisdiamine exposure in mice.^30^ No other gross abnormalities were observed.

**Table 2:** A mouse model of teratogen-induced CDH: descriptive characteristics.

Litters examined (*n*)	10
Implantations observed (*n*)	85
Fetuses examined (*n*)	53
Resorptions (*n*)	32
Resorptions (%)	37.6%
Diaphragmatic hernia (*n*)	26
Diaphragmatic hernia (%)	49.1%

### Altered maternal vitamin A status impacts susceptibility to teratogen-induced CDH

**Fig. 3:**
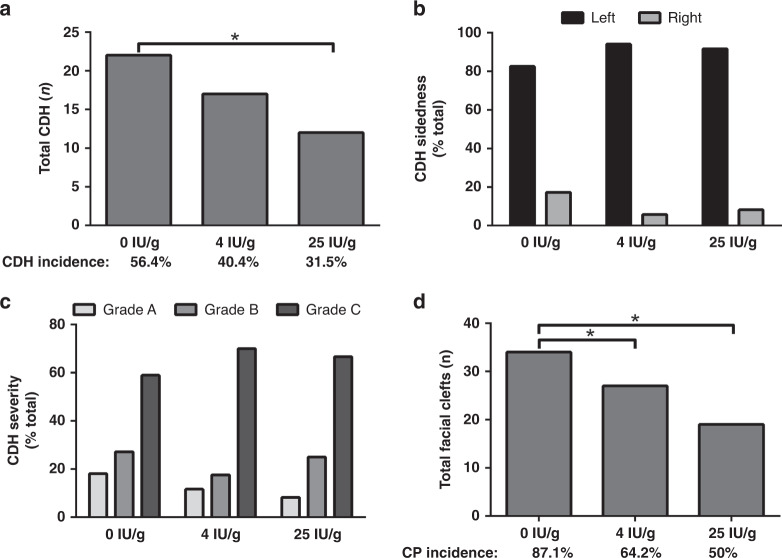
Altered maternal vitamin A status impacts susceptibility to teratogen-induced CDH. The total number of diaphragmatic hernia was significantly different between groups with differing vitamin A status (**a**). Mice consuming a diet with 0 IU/g vitamin A had a significantly higher incidence of CDH than mice consuming a 25 IU/g vitamin A diet. There was no significant difference in the sidedness (**b**) or severity of diaphragmatic hernia (**c**). The occurrence of midline facial clefting was significantly different between groups with differing vitamin A status (**d**). Mice consuming a diet with 0 IU/g vitamin A had a significantly higher incidence of facial clefting than mice consuming 4 or 25 IU/g vitamin A diet. All categorical data analyzed using a *X*^2^ test for trend. *p < 0.05, Fishers exact test post-test analysis.

Having validated the ability to alter maternal vitamin A status by manipulating dietary vitamin A intake, and establishing a teratogenic mouse model of CDH, we next tested the effect of maternal vitamin A status on the susceptibility to teratogen-induced CDH. As described in Table 3, we examined the incidence of CDH in teratogen-treated mice consuming diets with 0 IU/g vitamin A (12 litters), 4 IU/g vitamin A (13 litters), and 25 IU/g vitamin A (11 litters), including a total of 119 fetuses. In agreement with our hypothesis, there was a significant relationship between maternal vitamin A status and the number of diaphragmatic hernia observed, *Χ*^2^ (1, *N* = 119) = 4.859, *p* = 0.028 (Fig. 3a). Pair-wise analysis of each subgroup showed a significant difference between mice consuming 0 IU/g vitamin A and mice consuming 25 IU/g vitamin A, with the highest occurrence of CDH observed in the group consuming no vitamin A. No effect of maternal vitamin A status was observed for the side of the diaphragm defect (Fig. 3b), which occurred on the left-hand side in 80–90% of fetuses. Similarly, no effect of maternal vitamin A status was observed for the severity of the diaphragm defects (Fig. 3c), with 60–70% of hernias observed within grade C. Similar to the occurrence of diaphragm defects, we did observe a significant relationship between maternal vitamin A intake and the occurrence of facial clefting, *Χ*^2^ (1, *N* = 119) = 12.10, *p* = 0.0005 (Fig. 3d). Pair-wise analysis of each subgroup showed a significant difference between mice consuming 0 IU/g vitamin A and mice consuming 4 and 25 IU/g vitamin A, with the highest occurrence of facial clefting observed in the group consuming no vitamin A. We observed no significant effect of maternal vitamin A intake on the incidence of fetal oedma.

**Table 3:** Altered maternal vitamin A intake and status impacts susceptibility to teratogen-induced CDH.

	Experimental group		
	0 IU/g vitamin A	4 IU/g vitamin A	25 IU/g vitamin A
Maternal age (days)*	174 ± 30.7^a^	164.8 ± 27.5^ab^	145 ± 14.7^b^
Crown–rump length (mm)*	15.5 ± 2.4	15.9 ± 1.1	15.2 ± 1.2
Litters (*n*)	12	13	11
Litter size (*n*)*	8.9 ± 1.2	8.5 ± 1.6	8.8 ± 2.4
Fetuses (*n*)	39	42	38
Resorptions (*n*)^#^	68	68	59
Resorptions (%)	63.6%	61.8%	60.8%
Diaphragmatic hernia (*n*)^#^	22^a^	17^ab^	12^b^
Diaphragmatic hernia incidence (%)	56.4%	40.4%	31.5%
Facial cleft (*n*)^#^	34^a^	27^b^	19^b^
Facial cleft incidence (% total)	87.1%	64.2%	50.0%
Edema (*n*)^#^	11	7	7
Edema incidence (% total)	28.2%	16.6%	25.9%

*Continuous data are presented as the mean ± SD and analyzed by one-way ANOVA. ^#^Categorial data analyzed using a *X*^2^ test for trend. For both analyses, groups with the same superscript letter are not significantly different and groups with different superscript letters are significantly different.

## Discussion

The retinoid hypothesis proposes that abnormal retinoid signaling leads to abnormal diaphragm embryogenesis and causes CDH.^3^ Recent epidemiological data have suggested that low maternal vitamin A intake during pregnancy is a risk factor for CDH.^26,27^ With this in mind, the major goal of this study was to test the hypothesis that low dietary maternal vitamin A intake increases the risk of teratogen-induced CDH in mice.

To test this hypothesis, we had to develop and validate a model of altered maternal vitamin A intake and its effect on maternal and fetal vitamin A status. Following a 3-month dietary intervention, we established that female mice consuming a diet with no vitamin A for 3 months had a marginal vitamin A status, as evidenced by reduced hepatic retinoid stores. Female mice in our sufficient and excess vitamin A groups had hepatic retinoid stores that matched their dietary vitamin A intake level. It is important to emphasize that female mice consuming a diet with no vitamin A had a marginal vitamin A status but were *not* vitamin A deficient. These mice maintained residual hepatic retinoid stores and their circulating retinol levels were not significantly different than mice with a sufficient vitamin A status. This distinction is important because complete vitamin A deficiency has been shown to cause CDH in rats.^8–10,18^ In our control experiment we established that pregnant mice with a marginal vitamin A status did not spontaneously develop CDH or any other gross abnormalities. Similarly, while too much vitamin A can be teratogenic,^39^ our dietary intervention study with 25 IU vitamin A/g is within the physiological range and no gross abnormalities were observed in control litters in this group. Thus, while our dietary vitamin A intervention was enough to modulate maternal vitamin A status, it was not teratogenic.

The predominant animal model of CDH is the rat nitrofen model.^28,29^ As highlighted by Beurskens et al.,^28^ it is difficult to induce CDH in mice with nitrofen; however, we chose a mouse model for the ease of dietary vitamin A manipulation and to facilitate future research in transgenic mice, paving the way for a genetic dissection of CDH not possible in rats. To circumvent this limitation, we modified a teratogenic combination of nitrofen and bisdiamine in mice that has previously been described in limited studies.^17,30^ We optimized this teratogenic combination for mice achieving a CDH incidence of ~50%. This rate of CDH incidence was considered optimal for our dietary intervention studies, where an increased or decreased occurrence of CDH was possible. Phenotypically, this model produced diaphragm defects consistent with human Bochdalek CDH, including a complete hole in the posterolateral region of the diaphragm, herniation of abdominal organs into the thoracic cavity, and visible compression of the lungs. As a known abortifacient,^40^ bisdiamine is likely responsible for the relatively high resorption rate we observed, yet inclusion of this teratogen seems necessary to reliably induce CDH in mice. Indeed, in our preliminary studies it was the dose of bisdiamine that we titrated down to balance CDH incidence with resorption rate.

Having developed our model of dietary vitamin A intervention and optimized our mouse model of teratogen-induced CDH, we uniquely applied this experimental model to test our hypothesis that maternal vitamin A status impacts the susceptibility to CDH. Our data clearly show that the incidence of teratogen-induced CDH varied with maternal vitamin A intake and status, with a significant difference between animals with a marginal vitamin A status (56.4% CDH) and excess vitamin A status (31.5% CDH). Surprisingly, we observed no effect on the severity of CDH. In terms of sidedness of the observed defects, the group with marginal vitamin A status had a slightly higher occurrence of right-sided diaphragm defects, which are associated with a worse prognosis in humans,^41^ but this difference was not significant. Similarly, in terms of the size of diaphragm defects observed there was no effect of maternal vitamin A status. As to why marginal vitamin A status might impact the susceptibility to teratogen-induced CDH, our data show that fetal vitamin A status is affected by maternal diet. Thus, low fetal vitamin A status may make the developing diaphragm more susceptible to teratogen-induced perturbations in the retinoic acid signaling pathway, and higher vitamin A status may protect against these disturbances.

Our results show that marginal maternal vitamin A status increases the incidence of teratogen-induced CDH in mice. It is important to emphasize that we undertook a dietary manipulation of maternal vitamin A intake, creating three groups of mice with marginal, sufficient, and excess vitamin A status. We believe this nutritional approach speaks to the human epidemiological data showing that low maternal vitamin A intake during pregnancy increases risk of CDH.^26,27^ Moreover, our nutritional approach is distinct from previous studies in rats that demonstrated co-administration of nitrofen and large doses of vitamin A could prevent the development of CDH.^13,14,21^ In these rescue experiments, bolus doses of vitamin A (all-*trans*-retinol) were given at a concentration of 15,000–25,000 IU vitamin A. This represents a large pharmacological dose that is 1.5–2.5 times greater than the upper limit of recommended dietary vitamin A intake during pregnancy (10,000 IU/day,^42^ without even considering dose equivalencies between rats and humans. On the other hand, our dietary manipulation included a maximum amount of 25 IU vitamin A/g of diet, which is within the physiological range and not considered teratogenic. Thus, our data show that modulating maternal dietary vitamin A changes maternal vitamin A status and alters susceptibility to teratogen-induced CDH.

This study and the existing epidemiological data^26,27^ suggest that in the context of low vitamin A intake during pregnancy, women may be susceptible to other insults leading to CDH that could be environmental or genetic in origin. Strikingly, an analysis of usual dietary intakes of nutrients in pregnant women has shown that ~15% of women have a dietary vitamin A intake less than the EAR (estimated average requirement), representing a possible at-risk population for the development of CDH.^43^ In this context, there is a need for further research into the importance of vitamin A intake during pregnancy and preconception. The studies by Beurskens et al.^27^ and Michikawa et al.^26^ that found an association with low vitamin A intake and increased risk of CDH surveyed the maternal diet during pregnancy. Previous analyses of data from the National Birth Defects Prevention Study (1997–2003) showed that low intake of retinol in the year prior to conception was associated with an increased incidence of CDH in women not taking a nutritional supplement (odds ratio = 2.1 [95% CI 1.1–3.9]),^22^ highlighting the possible importance of maternal vitamin A intake prior to pregnancy as well. However, a more recent analysis of the National Birth Defects Prevention Study (1997–2011) did not find an association between dietary vitamin A intake and CDH.^44^ Thus, there are outstanding questions regarding the importance of vitamin A intake prior to conception, as well as during pregnancy that require resolution.

While the major focus of these studies was on the occurrence of CDH, we also recorded the effects of teratogen treatment and maternal vitamin A status on two other gross abnormalities: facial clefting and edema. The co-occurrence of facial clefting, edema, and diaphragm defects have previously been reported following teratogen treatment,^30^ and pharmacological inhibition of retinoic acid signaling in mice.^17^ The occurrence of facial clefting is consistent with the description of compound retinoic acid receptor mutant mice and established links between retinoic acid signaling and craniofacial morphogenesis.^45–48^ In addition to a modified susceptibility to CDH, our data clearly show that maternal vitamin A status has an impact on teratogen-induced facial dysmorphogenesis. Interestingly, this finding is consistent with epidemiological data showing that maternal vitamin A intake is associated with a reduced risk of cleft palate.^49^ The presence of generalized edema in CDH is thought to be secondary to obstruction of venous return to the heart and increased systemic venous pressure,^50^ with heart abnormalities also being a frequent comorbidity of CDH.^51^ The occurrence of edema in our animal model may reflect the known role of retinoic acid signaling in heart development;^52^ however, we did not find a significant effect of maternal vitamin A status on this particular abnormality. Beyond our own study, it was recently reported that maternal vitamin A intake can modulate the phenotype of *LgDel* mice, a model of 22q11.2 deletion syndrome;^53^ thus, there are other examples in the literature highlighting the importance of maternal vitamin A intake in the context of other birth defects.

Human epidemiological studies have suggested that low maternal vitamin A intake increases the risk of CDH.^22,26,27^ Our novel animal study confirms this association and shows that low dietary vitamin A intake and marginal maternal vitamin A status increases the incidence of teratogen-induced CDH in mice. The implications of this research are twofold. Low dietary vitamin A intake and marginal vitamin A status increases the susceptibility to CDH, whereas increasing maternal vitamin A intake at nutritional levels may lower CDH risk. The known teratogenicity of excess vitamin A intake warrants caution,^54^ but further research is required to determine if public health measures to ensure that pregnant mothers meet their daily recommended intake of vitamin A may lower the incidence of CDH (and possibly other birth defects) at the population level.

There are some limitations to this study. In our animal study we did not systematically analyze lung tissue in our experimental mice. This is an important future direction because retinoid signaling is also important in the developing lung and altered retinoid signaling in this tissue may represent a “second-hit” in addition to compression from abdominal organ herniation.^5–7^ We also limited our analysis of maternal vitamin A intake to a teratogenic model of CDH, but in the future it would be worthwhile testing whether maternal vitamin A intake can modulate the phenotype of genetic models of CDH, which would suggest that a combination of genetic factors and impaired vitamin A status combine to increase CDH risk.

In conclusion, using an animal model, this work supports recent epidemiological studies linking maternal dietary vitamin A intake and CDH risk and shows for the first time that inadequate vitamin A intake in mice increases the susceptibility to teratogen-induced CDH in mice. This work provides further support for the retinoid hypothesis of CDH etiology and highlights the importance of further research into the etiologic and pathogenic role of altered retinoid signaling in CDH.
